# A Zebrafish/Drosophila Dual System Model for Investigating Human Microcephaly

**DOI:** 10.3390/cells11172727

**Published:** 2022-09-01

**Authors:** Slawomir Bartoszewski, Mateusz Dawidziuk, Natalia Kasica, Roma Durak, Marta Jurek, Aleksandra Podwysocka, Dorothy Lys Guilbride, Piotr Podlasz, Cecilia Lanny Winata, Pawel Gawlinski

**Affiliations:** 1 Department of Biology, Institute of Biology and Biotechnology, University of Rzeszów, 35-601 Rzeszów, Poland; 2 Department of Medical Genetics, Institute of Mother and Child, 01-211 Warsaw, Poland; 3 Department of Animal Anatomy, Faculty of Veterinary Medicine, University of Warmia and Mazury in Olsztyn, 10-719 Olsztyn, Poland; 4 Independent Researcher, Manhiça MPT 1122, Mozambique; 5 Department of Pathophysiology, Forensic Veterinary Medicine and Administration, Faculty of Veterinary Medicine, University of Warmia and Mazury in Olsztyn, 10-719 Olsztyn, Poland; 6 Laboratory of Zebrafish Developmental Genomics, International Institute of Molecular and Cell Biology in Warsaw, 02-109 Warsaw, Poland

**Keywords:** microcephaly, zebrafish, *Drosophila*, microtubules, *TUBGCP2*

## Abstract

Microcephaly presents in neurodevelopmental disorders with multiple aetiologies, including bi-allelic mutation in *TUBGCP2*, a component of the biologically fundamental and conserved microtubule-nucleation complex, γ-TuRC. Elucidating underlying principles driving microcephaly requires clear phenotype recapitulation and assay reproducibility, areas where go-to experimental models fall short. We present an alternative simple vertebrate/invertebrate dual system to investigate fundamental *TUBGCP2*-related processes driving human microcephaly and associated developmental traits. We show that antisense morpholino knockdown (KD) of the *Danio rerio* homolog, *tubgcp2*, recapitulates human *TUBGCP2*-associated microcephaly. Co-injection of wild type mRNA pre-empts microcephaly in 55% of KD zebrafish larvae, confirming causality. Body shortening observed in morphants is also rescued. Mitotic marker (pH3) staining further reveals aberrantly accumulated dividing brain cells in microcephalic *tubgcp2* KD morphants, indicating that *tubgcp2* depletion disrupts normal mitosis and/or proliferation in zebrafish neural progenitor brain cells. *Drosophila melanogaster* double knockouts (KO) for *TUBGCP2* homologs *Grip84/cg7716* also develop microcephalic brains with general microsomia. Exacerbated *Grip84/cg7716-*linked developmental aberration versus single mutations strongly suggests interactive or coinciding gene functions. We infer that *tubgcp2* and *Grip84/cg7716* affect brain size similarly to *TUBGCP2* and recapitulate both microcephaly and microcephaly-associated developmental impact, validating the zebrafish/fly research model for human microcephaly. Given the conserved cross-phyla homolog function, the data also strongly support mitotic and/or proliferative disruption linked to aberrant microtubule nucleation in progenitor brain cells as key mechanistic defects for human microcephaly.

## 1. Introduction

Microcephaly is a neurodevelopmental condition occurring in 2–3% of the population [1]. Clinically, patients present with a head circumference less than minus two standard deviations of the normal sex and age matched average, reduced brain volume, and a high incidence of intellectual deficiency. Developmental delays and other developmental anomalies also seen with variable penetrance in other conditions and syndromes are common with microcephaly presentations [2,3,4]. Specific aetiology for microcephaly is therefore often indistinguishable at the clinical level. Environmental triggers include viral infections or chemical exposure in utero or during early childhood development, but most microcephaly is genetic [5,6]. Over 800 loci, encompassing the full range of mutation types, including polyploidy, are linked to microcephaly [7]. The molecular and cellular mechanisms directly affecting the development of microcephaly, however, remain imperfectly understood and, given the extensive aetiology, might appear to be many and varied. At first glance, untangling these seems to be a daunting task.

Bi-allelic mutation in any of at least 20 human genes, including *TUBGCP2* [8], causes autosomal recessive primary microcephaly (MCPH); most of these genes encode microtubule or microtubule-associated proteins [4,5,9]. Microtubules are formed from heterodimers of α- and β-tubulin isoforms at cellular microtubule organizing centres (MTOCs). Centrosomes, which orchestrate cell division, are the major MTOCs in vertebrate cells [10]. MTOC position and microtubule-nucleating activity within the cell are both controlled by the conserved eukaryotic microtubule-nucleating γ-tubulin ring complex, γ-TuRC. This complex in turn consists of multiple γ-tubulin small complex (γ-TuSC) subunits. One γ-TuSC subunit contains two γ-tubulin molecules complexed to one γ-tubulin-complex protein 2, (TUBGCP2) and one γ-tubulin-complex protein 3, (TUBGCP3). In higher eukaryotes, including humans, γ-TuSC subunits include a wider range of tubulin-gamma-complex proteins, TUBGCP 4, 5, and 6, [9,11], suggesting a degree of overlapping or exchangeable functional capacity.

Microtubules and microtubule-associated proteins such as TUBGCP2 are therefore integral to all fundamental molecular and cellular processes of early development [3,4,9,12,13]. This includes chromosome separation, centrosome, centromere, centriole, and spindle dynamics required for viable symmetric or asymmetric cell division [14,15,16,17,18], cellular proliferation, differentiation, and migration [3,4,19,20,21]. Microtubules and associated proteins are also integral to cytoskeletal structure, organelle positioning, molecular transport, tracking, and compartmentalization [9]. These basic molecular machineries are all tightly conserved throughout eukaryotic cellular biology [12,22,23,24,25]. They are fundamental to the precise spatial and temporal orchestration of progenitor cell proliferation, distribution, zone differentiation, and neuromigration processes of early brain development in vertebrates [9].

The fundamental nature of these machineries means that participating molecules will have extensive first, second, third, and more levels of interacting partners throughout development. This background provides for a scenario where myriad and apparently disparate functions along the interconnected developmental pathways and timelines, disrupted by mutation, will each ultimately cause a similar developmental failure, with a core differentiation endpoint phenotype, in this case, microcephaly. Therefore, to facilitate investigation of the most basic underlying mechanisms of microcephaly, we use the simplest possible, best-developed vertebrate and invertebrate in vivo neurodevelopmental research systems with discernible brains, viz., the zebrafish model complemented by the fruit fly system. In these, we examine cellular and phenotypic effects of depleting the respective homologs of *TUBGCP2*, a constituent of the most fundamental of cellular machineries, for which bi-allelic mutations in humans results in microcephaly [4].

## 2. Materials and Methods

### 2.1. Maintenance of Zebrafish

Wild type zebrafish were maintained in the zebrafish facility of the International Institute of Molecular and Cell Biology in Warsaw (license no. PL14656251), according to standard procedures and ethical practices. Adult fish were maintained at 28.5 °C with a 14 h light/10 h dark cycle. Larvae were grown in larvae medium at 28.5 °C and larval stages were determined by the number of hours post-fertilization (hpf) and microscopic observation of gross morphology [26].

### 2.2. MO Microinjection of Zebrafish Single Cell Stage

Two antisense MO for zebrafish tubulin-gamma-complex associated protein 2 (tubgcp2 (NM_200122.1)) sequences were designed by the manufacturer to target the 5′ UTR region before the start codon:

(tubgcp2_transblock: CGTAAAGCATCTGTTGTAGAAGAGT)

and the splice site between exons 7 and 8:

(tubgcp2_splice_e7i7:TGCTGATATCTTTACCAATGGAGGC) (GeneTools. LLC, Philomath, OR, USA).

Subsequently, 4 ng of each MO plus 4 ng of p53 apoptosis suppression MO was injected into the yolk of single-cell stage larvae from wild type AB/TL zebrafish strains. Injected larvae were incubated at 28.5 °C until reaching the appropriate developmental stage. Consistent phenotype was observed in at least three independent experiments using 90–150 larvae each. mRNA rescue experiments used co-injection of 4 ng of either tubgcp2_transblock-MO or tubgcp2_splice_e7i7-MO, 4 ng of p53 apoptosis suppression MO, and 25 pg of tubgcp2 mRNA. The mRNA dosage was determined by injection of 25, 50, and 75pg of tubgcp2 mRNA into the yolk of single-cell stage embryos from wild type AB/TL zebrafish strains. A higher dosage resulted in a higher larval mortality rate and introduction of nonspecific morphological abnormalities. As a negative control, standard control oligo (https://store.gene-tools.com/content/standard-control-oligo) (accessed on 27 August 2022) was used.

### 2.3. Tubgcp2 mRNA Synthesis

Total RNA was extracted from 15 wild type AB/TL zebrafish larvae at various stages (at 1, 2, and 3 days post-fertilization (dpf)) using an RNAeasy Mini Kit (Qiagen, Germantown, MD, USA). A total of 0.25 μg of the resultant total RNA was used as a template for RT-PCR analysis using a High-Capacity cDNA Reverse Transcription Kit (Applied Biosystems, Waltham, MA, USA). The following primers were used to amplify full-length *tubgcp2:*

cDNA (NM_200122.1): zTUBGCP2-F 5′- ATGAGTGAATTCAGGATTCATCA-3′ and zTUBGCP2-R 5′- CTACGCTACCGTCTTCTGG -3′. Forward primer had T7 promoter sequence (5′-TAATACGACTCACTATAGGG-3′) on the 5′ end preceding the gene-specific sequence, which was used for capped tubgcp2 mRNA synthesis using a mMESSAGE mMACHINE T7 Transcription Kit (Invitrogen, Waltham, MA, USA), according to the manufacturer’s instruction. The reactions were then purified with the RNAeasy Mini Kit (Qiagen, Germantown, MD, USA). The translation-blocking MO targets the 5′ UTR region before the start codon; therefore, the synthesized wild type mRNA coding sequence does not require protective modification (normally achieved by introducing silent mutations).

### 2.4. Zebrafish Phenotype Analysis

Injected larvae incubated for 3 dpf were examined under a TL5000 Ergo (Leica, Wetzlar, Germany) stereomicroscope. Larvae were photographed at dorsal view. Body length and head width of larvae were measured using Fiji image analysis software (v. 2.6.0; Zurich, Switzerland) [27].

### 2.5. Whole-Mount Immunohistochemistry for Zebrafish Assays

Immunolabelling of Tg(neuroD:GFP) larvae 2 dpf was completed according to Podlasz et al. 2016 [28]. Briefly, larvae at 2 dpf were fixed with 4% PFA overnight (o/n) at 4 °C. Specimens were washed in PBS with 0.3% Triton X-100 (PBST; pH 7.4) three times for 30 min, with gentle shaking. Specimens were then set in blocking solution containing PBST with 1% dimethyl sulfoxide (DMSO), 4% normal goat serum (NGS), and 0.1% sodium azide o/n at 4 °C. Specimens were then incubated with anti-pH3 (Ser 10) primary antibody (1:5000) o/n at 4 °C with gentle shaking. Samples were then washed 3 × 30 min in PBST at room temperature and incubated with Rabbit IgG Alexa 555 secondary antibody (1:1000, Invitrogen, # A-21431, Waltham, MA, USA) o/n at 4 °C with gentle shaking. Specimens were then washed 3 × 30 min in PBST and 2 × 60 min in 50% glycerol/PBS, then mounted in 80% glycerol in PBS.

### 2.6. Microscopy and Quantification Assays for Zebrafish

Images were made using an LSM 700 confocal laser scanning microscope, ×10 objective (Zeiss, Oberkochen, Germany) with 488 nm and 555 nm lasers for GFP and pH3 (Ser 10) excitation, respectively. Compiled image stacks (ZEN 2009 software (Zeiss, Germany) were used to obtain maximum intensity projection. Ten images per group were analysed. Quantification (ImageJ software version 1.51j8, U. S. NIH, Bethesda, MD, USA) was derived from the forebrain and midbrain area within the white line along the midbrain-hindbrain border.

### 2.7. Drosophila Strains, Provenance, Mutant Generation, Maintenance, and Assays

Fly strain provenance: Mutants, *Grip84^PG36^* and *Grip84^PG66^*, and rescuing construct for *Grip84*, *P[Ub84,w+]*, were obtained from Henri-Marc Bourbon (Centre de Biologie du Développement, Université Paul Sabatier, Toulouse, France), deficiency *Df(1)14.4* from Veit Riechmann (Heidelberg University, Medical Faculty Mannheim, Department of Cell and Molecular Biology, Mannheim, Germany), and most of the other stocks from Bloomington Drosophila Stock Center, Department of Biology, Indiana University, Bloomington, IN, USA.

Fly mutant constructs: To obtain a convenient balancer rescuing *Grip84* activity, appropriate lines were crossed together to get male flies *CyO, H{PDelta2–3}HoP2.1/+;P[Ub84,w+]/TM6B, Tb e*. They were crossed individually to *w*; *sr e* female flies, and *e*, *w+* individuals found in progeny were tested for segregation with *TM6B*; one line with an insertion was found and used. *Dp(1;3)DC364, PBac{y+w+}* was recombined to *Mi{ET1}CG7716^MB07394^* in *y w* background, selecting for flies that were *w+* and expressed GFP in eyes and ocelli. The above lines were bred to get the stocks *w Df(1)14.4; Dp(1;3)DC364 PBac{y+w+}/TM6B, P[Ub84,w+]*, and *w Df(1)14.4; Mi{ET1}CG7716^MB07394^ Dp(1;3)DC364 PBac{y+w+}/TM6B, P[Ub84,w+]*. The *CG7716^MB07394^* flies are viable and fertile.

Fly maintenance: Flies were cultured on a standard yeast, glucose, and corn meal medium and experiments were carried out at 25 °C. For analyses of larvae, eggs were collected on apple juice and sucrose plates seeded with yeast. Two days after egg collection, a coffee spoon of pulp sedimented from homemade red wine and seeded with dry yeast was also added onto the plate. Guts of the larvae that foraged on the paste were intensively red until they stopped eating in the last 12 h before pupation. 

Fly histochemistry assays: Larvae were dissected in PBS buffer and brains were fixed in 3.7% formaldehyde in PBS for 20 min, then washed roughly with PBS and 3 times 10 min in PBT (0.1% Triton X-100 in PBS), followed by 30 min incubation in 1% BSA in PBT. After dissection, pupal brains were left overnight in the same formaldehyde and PBS solution at 4 °C, then processed in the same way. The brains were stained for 2 h with anti-phospho-histone H3 (Ser10) antibody, clone 3H10, Merck, diluted 5000 times, then washed 3 times 5 min with PBT and stained overnight at 4 °C with anti-mouse Alexa-568 antibody, followed by 3 times 10 min in PBT. DAPI was added to the last portion of PBT and kept overnight at 4 °C, then the brains were placed in PBT, 90% glycerol, and 0.02% sodium azide between two coverslips. Specimens were scanned on a LSM710 confocal microscope with 20× magnification. Brains were analysed with Zeiss Axiovision software (v. 4.9.1, Zeiss, Oberkochen, Germany); the central sections were outlined separately for each hemisphere and the software calculated the surface with about 15 images analysed for each group.

## 3. Results

### 3.1. Zebrafish Tubgcp2 Depletion Induces Microcephaly 

**Figure 1:**
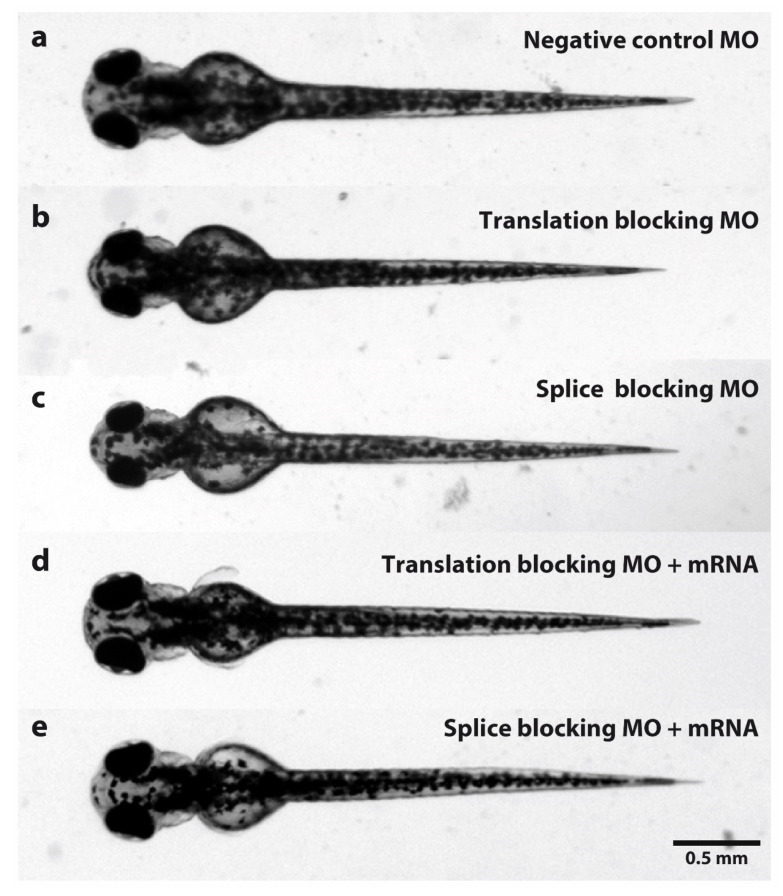
Dorsal view comparison of zebrafish larvae at 3 dpf injected with MOs. (**a**) Standard control oligo was injected as a negative control. (**b**) Zebrafish larvae injected with *tubgcp2* translation-blocking MO. (**c**) Zebrafish larvae injected with *tubgcp2* splice-blocking MO. (**d**) Zebrafish larvae co-injected with *tubgcp2* translation-blocking MO and *tubgcp2* wild type mRNA. (**e**) Zebrafish larvae co-injected with *tubgcp2* splice MO and *tubgcp2* wild type mRNA.

To examine the effect of *TUBGCP2* depletion on brain development, we used knockdown (KD) of the homolog *tubgcp2* in the zebrafish model. We microinjected single-cell-stage fish larvae with *tubgcp2*-specific morpholino antisense oligonucleotides (MOs), blocking either translation or splicing of *tubgcp2* transcripts in the presence of p53-blocking MO to exclude the possibility of MO-induced apoptotic effects. Representative phenotypes for control, morphants, and wild type mRNA-rescued forms are shown relative to each other in Figure 1.

**Figure 2:**
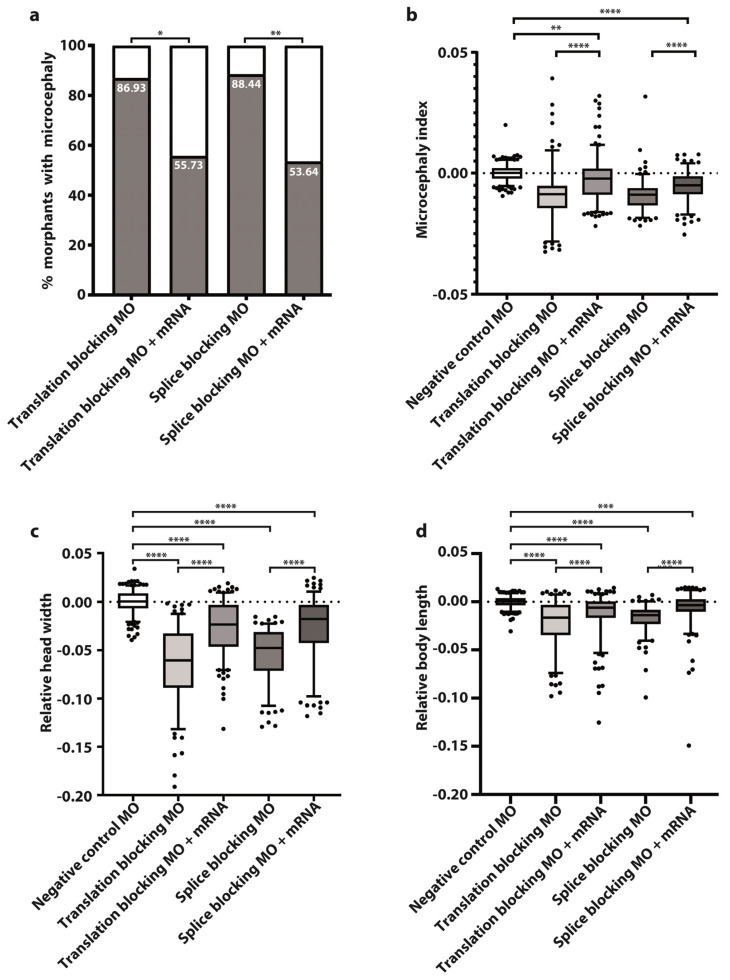
Wild type *tubgcp2* mRNA pre-empts microcephaly in *tubgcp2*-depleted morphants at 3 dpf. (**a**) Percent of morphant larvae obtained with microcephaly when treated with *tubgcp2* translation-inhibiting or splicing-inhibiting MO (see Supplementary Table S1 for experimental numbers and statistics). (**b**) Microcephaly index = head width/body length ratio = (**c**)/(**d**) for tubgcp2-depleted and *tubgcp2* mRNA-rescued morphants. (**c**) Head width; (**d**) body length. Statistical multiple comparisons completed using one-way ANOVA; * *p* ≤ 0.05, ** *p* ≤ 0.01, *** *p* ≤ 0.001, **** *p* ≤ 0.0001. This includes Ŝidák’s correction for multiple comparisons.

In larvae injected with splice- or translation-blocking MOs, an experimental average of 88% develop the microcephaly phenotype (defined as a reduction of head width by more than two standard deviations compared to controls) (Figure 2a). Morphant mutant phenotype is visually indistinguishable between KD classes (Figure 1b,c) with very similar morphant levels obtained in either class (86.93% and 88.44%, respectively) (Figure 2a). 

After co-injecting MOs with wild type tubgcp2 mRNA, an average of 55% developing larvae (55.75% of trans MO group; 53.64% of splice MO group) developed almost normal brain size (Figure 1d,e; Figure 2c,d). This indicates brain size is rescued by wild type tubgcp2 mRNA (Figure 2a). Both experimental groups generate very similar levels of rescue. The Microcephaly Index (head width to body length ratio; Figure 2b) uses head width and body length measures, shown in Figure 2c and Figure 2d, respectively. These data show *tubgcp2* depletion also causes a small but correspondent and significant decrease in body length (Figure 2d) accompanying microcephaly; *tubgcp2* mRNA co-injection therefore rescues both small brain size and shorter body length close to (i.e., within) normal values.

### 3.2. Tubgcp2 Depletion Disrupts Replicative and/or Proliferative Processes in Zebrafish Larval Progenitor Brain Cells 

**Figure 3:**
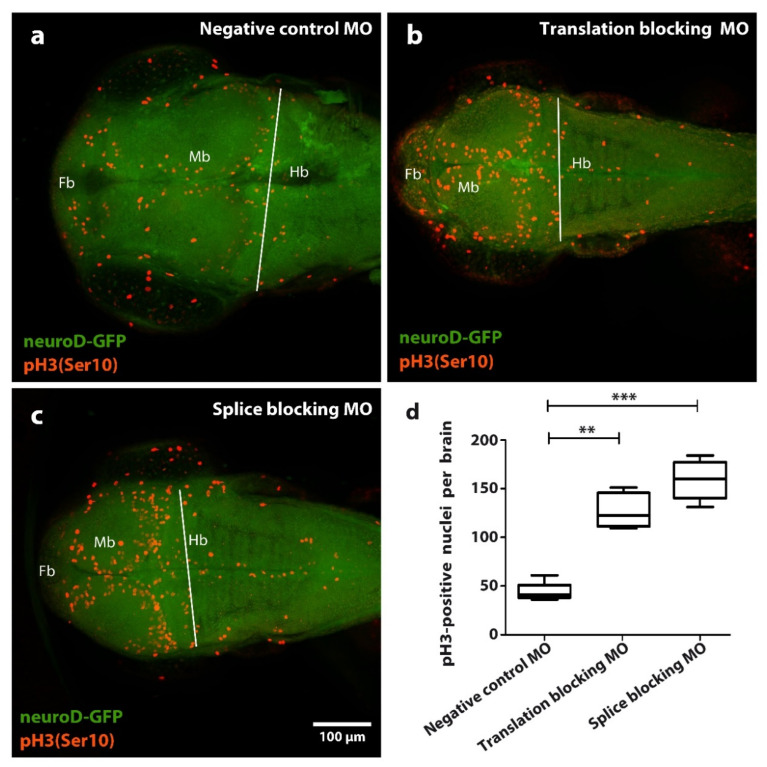
Effect of *tubgcp2* knockdown using microinjection of morpholino antisense oligonucleotides (MOs) blocking either splicing or translation, on number of pH3/Ser10) immunoreactive (IR) nuclei in zebrafish brain tissue at 3 dpf. Immunohistochemical staining of Tg(neuroD:GFP) larvae 2 dpf (green): pH3 (Ser 10) (red) shows distribution of pH3 (Ser 10) IR nuclei in (**a**) control, (**b**) translation-blocking MO, and (**c**) splice-blocking MO-treated groups within the forebrain (Fb), midbrain (Mb), and hindbrain (Hb). (**d**) Quantification of pH3 (Ser 10) IR nuclei in larvae at 2 dpf. Quantifications derive from Fb + Mb areas within limit (white line) lying along midbrain-hindbrain border. Statistical test one-way ANOVA and Kruskal–Wallis analysis was completed using GraphPad Prism 5; ** *p* ≤ 0.01, *** *p* ≤ 0.001. Compared to the control group (**a**), translation-blocking MO injection (**b**) results in an approximate 3-fold increase of pH3 (Ser 10) IR nuclei, whereas splice-blocking MO injection (**c**) results in a near 4-fold increase of pH3 (Ser 10) IR nuclei.

To assay mitotic cell activity in morphant and control group fish brains, we stained the brains in each group with the mitotic marker phospho-histone 3 (pH3) (Figure 3).

Compared to the control group (Figure 3a), we find significant and similar accumulation of mitotic brain cells in the morphant small-brain groups for both splicing and translation disruption (Figure 3b–d). However, these data do not distinguish over-proliferation from mitotic arrest. We therefore infer *tubgcp2* depletion causes disruption of either the mitotic cycle or proliferative process, or possibly both, in progenitor brain cells in the developing larvae brains. The distribution of the mitotic brain cells visibly reflects the underlying tissue organization of progenitor brain cell zones in the fish brain; an increased number of cells staining makes this organization more apparent in morphants.

### 3.3. Knock out of TUBGCP2 Fly Homologs Grip84/gcp7716 Causes Microcephaly in Drosophila Melanogaster

**Figure 4:**
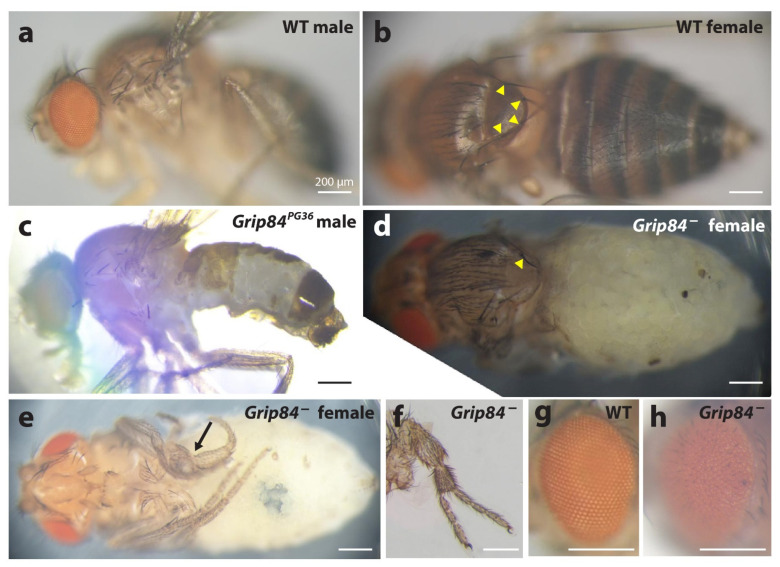
Phenotypes of the *Grip84* mutants. (**a**) Wild type (WT) male sibling and (**b**) (WT) female sibling, lateral and dorsal views, respectively. (**c**) *Grip84^PG36^* hypomorphic male fly lacking vast region of the normal cuticle on the abdomen. (**d**) Pharate adult female fly, *Df(1)14.4*; *Dp(1;3)DC364;* pulled out of the pupal case, shown from dorsal view and (**e**) ventral view. The abdominal pigmented cuticle is completely absent. Yellow arrowheads point at the bases of four bristles on the notum in the WT female fly; (**b**) in the mutant female fly, (**d**) only one bristle is left. In the mutant female, one of the legs is incorrectly developed ((**e**) arrow); in some cases partial leg duplications were observed for the same genotype (**f**). A wild type eye from panel **a** is magnified in (**g**), showing the characteristic, hexagonal structure, which is absent in *Df(1)14.4*; *Dp(1;3)DC364* flies (**h**).

Our Blast searches reveal two *Drosophila* homologs for *TUBGCP2*: *Grip84* and *cg7716* (Supplementary Figure S1). *Grip84* is the closest fly homologue for *TUBGCP2* with 37% and 59% amino acid identity and similarity, respectively. We examined *Grip84* mutants first described in earlier work [29]. We found these were semi-lethal and that male flies were sterile; however, escaper female flies produced progeny. Using available fly stocks with deficiencies and duplications, we obtained a mutant devoid of about half of the *Grip84* coding region and polyadenylation sequences (see Section 2 and Supplementary Figure S1a). These flies were more severely affected than previously described mutants and the few most developmentally advanced specimens obtained died during eclosure. Both of these developmentally advanced forms and the more frequently obtained pharate-stage adults showed new specific phenotypes (Figure 4): lack of bristles on the notum, severe lack of abdominal cuticle, improper leg development including axis duplication, and rough eyes.

**Figure 5:**
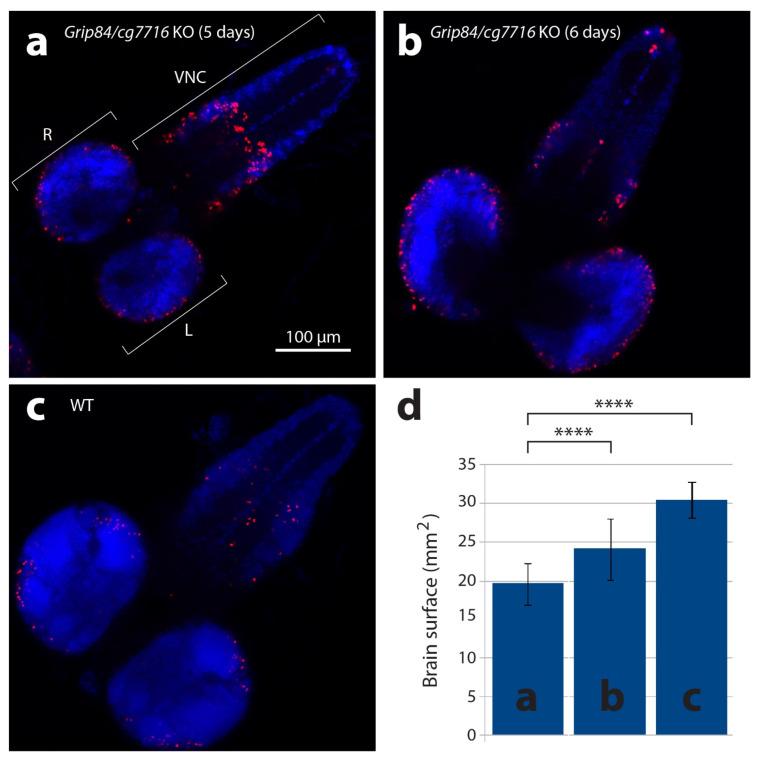
Larval brains stained with anti-phospho-histone H3 antibodies detecting mitotic nuclei (red) and DAPI. (**a**) Brain from five-day-old mutant larvae *Df(1)14.4; Mi{ET1}CG7716^MB07394^ Dp(1;3)DC364 PBac{y^+^w^+^}*, amorphic (activity absent) for both *Grip84* and *cg7716*. (**b**) Mutant six-day-old larvae brain and (**c**) five-day-old WT sibling larvae brain. Specimens shown in (**b**,**c**) are just before pupation. (**d**) Brain hemisphere surface area (SA) quantification for brains shown in (**a**–**c**), with standard deviation. Statistical multiple comparisons completed using one-way ANOVA; **** *p* ≤ 0.0001. R—right hemisphere, L—left hemisphere, VNC—ventral nerve cord.

The transgenic fly construct (see Section 2) completely rescues viability and other *Grip84* phenotypes. However, *Drosophila* has another, less conserved homologue, cg7716 (27/46 identity/similarity). To assess the effect of full depletion of *TUBGCP2* activity in the fly, we therefore created a double mutant knock out (KO) fly stock, knocking out expression of both *TUBGCP2* fly homologs, *Grip84* and *cg7716* (see Section 2 and Supplementary Figure S1). The *cg7716* mutation is viable and fertile. We found *Grip84*/*cg7716* KO larvae pupate at day 6, a day later than wild type (WT) siblings (day 5). Mutant larval brains at day 5 and 6 (Figure 5a,b) are markedly smaller than WT brains at day 5 (Figure 5c,d) and seem to be structurally abnormal.

**Figure 6:**
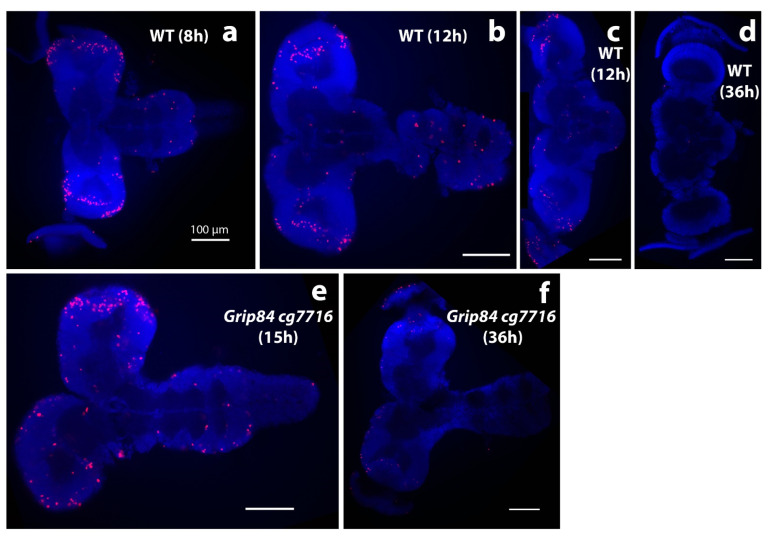
Brain morphology during pupation stained with DAPI stain (blue); anti-phospho-histone H3 (anti-pH3, red). (**a**–**d**) Brains from wild type siblings’ pupae; (**e,f**) *Df(1)14.4; Mi{ET1}CG7716^MB07394^ Dp(1;3)DC364 PBac{y^+^w^+^}* mutants. (**a**) Normal pupal brains 8 h after pupal case formation resemble larval brains, although the hemispheres elongate distally and show clear mitotic zones at the tips, marked by anti-pH3. (**b**) Further elongation is seen at 12 h, when normal brain and ventral nerve cord lose tight connection. (**c**) The majority of normal brains are dissected separately at this time point. (**d**) WT brains one day later at 36 h look different; the connection to eyes becomes visible; mitoses are not seen. Early stage mutant brains resemble wild type. (**e**) Mutant brains at 15 h (after early adult body parts are formed in normal pupae) and (**f**) at 36 h resemble “early 12 h wild type”. At 36 h, brain mitoses are barely detected. White bar = 100 μm.

Qualitatively, larval mutant microcephalic brains also appear to show accumulation of mitotic brain cells (compare Figure 5a,b to Figure 5c), although statistical analysis in this case is difficult to carry out due to problems with identical positioning of the objects in microscopic preparations. Surface area (SA) quantification (Figure 5d) shows that day 5 mutant fly brain SA is 64% of WT, and reaches 79% of WT brain SA just before mutant pupation, at day 6. Twelve hours after pupation, metamorphosis of imaginal discs is clearly seen in WT siblings but not in mutants, suggesting development is generally delayed in *Grip84/gcp7716* mutants. During 8–12 h, WT pupal brains then elongate, losing the strong connection to the ventral nerve cord (Figure 6a–c); at 36 h post-pupation, the connection of the WT brain to the developing eyes, established at the larval stage, becomes visible in the pupal brain (Figure 6d).

Early morphological development in post-pupation mutant brains appears to proceed, although apparently structurally abnormal, at a rate similar to WT until about 7–10 h post-pupation. However, mutant brains at 15 h still morphologically resemble “early 12 h” WT siblings, with brains closely connected to ventral nerve cords (VNC, compare Figure 6e to Figure 6b,c). At 12 h post-pupation, WT brains (Figure 6b) and mutant brains at 15 h (Figure 6e) also seem to have similar numbers and distribution of mitotic brain cells (Figure 6b,e). Therefore, it appears that mutant brain development may have stopped at around 12 h post-pupation; mutant brains at 15 and 36 h (Figure 6e,f) look morphologically and developmentally similar, except that the mitotic brain cells seen at 15 h (Figure 6e) are no longer visible at 36 h in mutant brains (Figure 6f). This is most likely because mutant pupae at 36 h are dead.

## 4. Discussion

In this work, we present a dual simple-vertebrate-invertebrate experimental model comprising the zebrafish, *Danio rerio*, and the fruit fly, *Drosophila menagaster,* as an alternative, if perhaps non-intuitive, approach to investigating human neurodevelopmental disease and microcephaly in particular. We show that two evolutionarily distant systems can be effectively used to examine basic molecular, cellular, and developmental impact arising from the disruption of biologically fundamental and highly conserved molecular machineries associated with human microcephaly, in this case, the microtubule–nucleation complex.

Assay for phenotype followed by wild type expression rescue is a broadly applicable first step in the investigative process for establishing gene function. The go-to in vivo mouse and in vitro stem-cell-derived brain organoid experimental systems, subject to research purpose, are indisputably valuable standard models in themselves. However, both have limitations with respect to effective recapitulation of microcephaly [30,31,32] and, therefore, in examining neurodevelopmental syndromes where microcephaly is a trait. There are previously reported difficulties with variable discernability of microcephaly in both mouse and organoid systems [30,31,32], and limitations as to developmental capacity and environmental and physiological input influence factors and physiological complexity in brain organoids [31,32]. Furthermore, “…the self-organizing nature of organoid structures and morphogenic heterogeneity introduces intrinsic heterogeneity and inherent stochasticity” [31,32] or, in other words, systemic difficulties with reproducibility. This means large numbers of specimens, painstakingly examined, often via immunohistochemistry, are required to obtain reproducible results in organoid-based assays. However, organoid generation requires large culture volumes. Reagent and space requirements therefore make routine laboratory scaleup of organoid specimen production a non-trivial hurdle [31,32]. Overall, these considerations make the mouse and organoid models, particularly with respect to phenotypic assay, reproducibility, interpretation, and handling issues, less experimentally convenient and efficient for many aspects of first-level microcephaly research.

We show that depletion of the *TUBGCP2* homologs, *tubgcp2* in the zebrafish and *Grip 84/cg7716* in the fly, both evolutionarily distant organisms, effectively recapitulates the microcephaly trait present in patients with bi-allelic *TUBGCP2* mutation. Both systems also recapitulate some developmental impact, with developmental delay and defective morphology most strongly apparent in the fruit fly system. Microcephalic zebrafish larvae treated with *TUBGCP2*–mRNA-specific MOs also display a slight but significant reduction in body length (Figure 2d), correspondent to the degree of microcephaly developed (Figure 2c), reflected in the Microcephaly Index (Figure 2b). Both microcephaly and short-body phenotypes (Figure 1b,c and Figure 2a–d) induced by MO knockdown of *tubgcp2* translation or splicing, are reproducibly pre-empted by co-injection of nonengineered WT *tubgcp2* mRNA, in 55% of the co-injected larvae (Figure 2a,b; Supplementary Table S1). This indicates that *tubgcp2* expression is required for both normal development of the brain and development of full body length. Both morphant traits, in equivalent numbers for both splice and translation knockdown groups, develop to almost normal measures when rescued with wild type *tubgcp2* mRNA, confirming causality for both traits. 

We conclude that *tubgcp2* depletion drives the microcephaly phenotype and also has a slight microsomal or inhibitory effect on general growth and development in the zebrafish. At the cellular level, examination of three-day-old morphant brains stained with the mitotic marker pH3 reveals accumulation of dividing brain cell progenitors. This is reminiscent of *tubgcp3* depletion in the zebrafish, which causes accumulating dividing forms without progression through cell division. In this case, *tubgcp3* depletion freezes mitosis in zebrafish retinal progenitor cells, resulting in abortive cellular proliferation, leading to a small-eye phenotype [19]. Our data strongly suggest that disruption of normal microtubule-nucleating functions requiring TUBCGP2 causes defective cell division and/or proliferative processes in progenitor brain cells, but do not distinguish between mitotic arrest, over-proliferation, or temporal derangement of the cell cycle. Nonetheless, the data clearly pinpoint involvement of a mechanism fundamental to cellular replicative mechanisms. This highlights the applicability of the system to investigations of fundamental molecular and cellular mechanisms associated with microcephaly.

Mechanistic, temporal, and spatial disturbance of cell division, cellular proliferation and migration processes, or premature differentiation of progenitor cells resulting in senescence, apoptosis, and depletion of progenitor cell pools have all been individually implicated or proposed as fundamental aberrations linked to autosomal recessive microcephaly [3,4,14,15,16,17,18,19,33,34]. For example, a recent study shows that depletion of the zebrafish homolog of microtubule associated protein MAP11, which interacts with α-tubulin at the centrosome, affecting cell proliferation, recapitulates the effect of bi-allelic human *MAP11* mutations resulting in microcephaly [3].

Therefore, it is reasonable to propose a scenario where the mitotic cell accumulations we observe in *tubgcp2*-depleted zebrafish progenitor brain cells, whether due to cell cycle disruption or proliferative derangement, leads to depleted progenitor cell populations, leaving insufficient cells to complete normal brain development, resulting in the microcephalic phenotype [3,35].

In the fly, the *Grip84/cg7716* double homolog KO results in clear microcephaly, but also general microsomia and strongly delayed development timeline. We also document several previously undescribed *Grip84*-specific single mutant aberrations in the development of the cuticle, bristles, torso, and leg (Figure 4). The surface area (SA) (Figure 5d) of mutant fly brains at day 5 is 64% the surface area of WT and reaches 79% just before pupation, at day 6. Development of the mutant pupae brains, although structurally abnormal, appears to continue for twelve hours after pupation, but then stops entirely; mutant pupae are dead at 36 h post-pupation. Evidently, development is delayed in larval *Grip84/cg7716* mutants until day 6 but totally stops 12 h later during pupal development. An excess of maternal *Grip84* mRNA carried over from the larval state, which runs out at this point in pupation, may explain this developmental profile.

With regard to a possible disruptive effect on mitosis by *Grip84/cg7716* KO in the fly, we comment that, qualitatively, we see an apparently increased number of mitotically dividing brain cells in pre-pupation mutant larvae relative to controls (Figure 5). This is qualitatively similar to our results in the mutant zebrafish brains. However, slide preparation for these fly brain images involves stretching out the fly brain tissue to flatten the tissue; this is not uniformly achieved. Quantitative assessment of mitotic staining cells in all focal planes is challenging and requires further analyses. We conclude that *Grip84*/cg7716 depletion in the fly, similarly to *tubgcp2* depletion in the zebrafish, results in microcephaly (Figure 5 and Figure 6) with marked new developmental defects observed (Figure 4) in our single mutant fly stock for *Grip84* depletion alone.

It is worth noting in this context that the specific defects previously reported for *Grip84* mutations in the fly are reduced viability and aberrations in larval brain cell division, with chromosome over-condensation or polyploidysation, as well as aberrations in meiotic cells leading to male fly sterility [29]. There is no record of microcephaly. Therefore, our results for the *Grip84*/cg7716 double KO mutant fly construct strongly suggest that *cg7716* mutation enhances *tubcgcp2* mutational impact to produce microcephaly. We infer that these two mutations interact and impact the same developmental pathway in brain cell replicative processes and brain size development, resulting in microcephaly. Mosaic experiments, a well-developed advantage of the fly system, may therefore be useful in resolving relative contributions to phenotype. Overall, the data available for *TUBGCP2* homolog disruption in the fly are consistent with our zebrafish findings of accumulating dividing progenitor cells, indicating either mitotic or proliferative disruption, or both. In both model systems *TUBGCP2* homolog disruption also has whole organism developmental impact, although less evident in the zebrafish.

The data in both model systems also directly link the function of the microtubule nucleation complex γ-TuRC, via component proteins, to brain development. Given the ubiquitous and fundamental function of this complex in all cell division underlying developmental differentiation throughout the organism, it is reasonable to expect microsomia and defective development in the rest of the organism.

This is, in fact, what we observe in the fly system: microcephaly accompanied by general microsomia and gross defects in developmental differentiation. Specific developmental defects observed, such as lack of bristles (Figure 4b,d), are reminiscent of phenotypes for mutations in the achaete–scute complex. This participates in neural cell development and interacts with the *Notch* pathway, hinting at interconnected or interacting pathways of neural development linked to *Grip84* and *cg7716* function. On the other hand, limb duplication (Figure 4f) might be connected to *Wingless* and/or *Hedgehog* activation. Defective development is less pronounced in the zebrafish, where, phenotypically at least, we see only slightly reduced body length apart from microcephaly. In human microcephaly patients carrying bi-allelic *TUBGCP2* (or other *TUBCGP*) mutations, developmental delays are usual, however anatomical defects, other than microcephaly, are less obvious and are generally more apparent on a cellular scale. An example is the defect in neuromigration leading to subtle structural defects in brain development, such as the lissencephaly (absence or diminishment of brain folds) that accompanies microcephaly with *TUBCGP2* mutation [33].

We cannot explain why bi-allelic mutations for *TUBGCP2* and *tubgcp2* do not result in general microsomia in the human or zebrafish; these results imply an evolutionary branchpoint where TUBGCP2 function still directly impacts cellular processes in brain development but is somehow buffered from general organismal development. We speculate that as the evolving organism becomes more highly differentiated during development, other factors (such as *TUBCGP* 3, 4, 5, 6), expression, stability and maternal mRNA interaction in early larval stages [19] in different tissues may normally replace, interact with, or compensate for defective components of the microtubule nucleation function. Our data in the fruit fly double mutant *Grip84/cg77166* support the notion that gene products interact with each other and influence the outcome of mutations in the *TUBGCP2* homologs along a developmental pathway. As noted, general developmental manifestation is much stronger for the amorphic (fully depleted) fruit fly *Grip84*/*cg7716* double and single KO mutants, relative to the *tubgcp2* KD zebrafish morphant. One possible reason for this may be that any KD is by definition a “leaky “ mutation, allowing some background function and, furthermore, the morpholino oligonucleotides (and therefore ongoing level of KD) are progressively diluted as zebrafish development proceeds. The more severe effects of total and sustained loss of function for *TUBGCP2* homologs are therefore more visible in the fruit fly. Counterintuitively, this implies that the invertebrate fruit fly system may prove to be the more useful tool for fundamental dissection of developmental aspects of the phenotype associated with vertebrate and human microcephaly. On the other hand, the zebrafish brain is more amenable to assays for the investigation of specific cellular processes such as mitosis, and tracking very early single cell developmental processes, since zebrafish larvae are transparent. Both are excellent systems for initial phenotype-based assays and scaleup relevant to pharmaceutical testing.

Overall, given the conserved participation of *TUBGCP2* homologs in molecular microtubule nucleating machineries, both our own data derived from the zebrafish and fruit fly systems and other available zebrafish data strongly support mitotic disruption and/or proliferative disorder of progenitor brain cells as key mechanistic defects linked to aberrant microtubule nucleation activities underlying human microcephaly. We conclude the zebrafish/fruit fly dual system that we present here provides a powerful alternative experimental system with a broad “fit for purpose” range and strong potential for facilitating conceptual and biomedical advancements for human microcephaly.

## Data Availability

Data are contained within the article.

## Supplementary Materials

The following are available online at https://www.mdpi.com/article/10.3390/cells11172727/s1, Table S1: Raw count and percentage of larvae with mutated or wildtype, Figure S1: Organisation of the Grip84 gene in *Drosophila melanogaster*.
