# Supplementary material for: A Zebrafish/Drosophila Dual System Model for Investigating Human Microcephaly
Source: Cells. 2022 Sep 1;11(17):2727. doi: 10.3390/cells11172727 (PMC9455030; doi:10.3390/cells11172727)
Supplement: Supplementary file 1 [file cells-11-02727-s001.zip › cells-1890396-supplementary.pdf]

Supplementary Table S1. Raw count and percentage of larvae with mutated (MUT) (defined as head width < -2SD below average) or wildtype (WT) phenotype. Statistical significance for microcephalic forms (MUT) obtained in KD vs RESCUE experiments: Multiple comparisons one-way ANOVA; * : P ≤ 0.05; **: P ≤ 0.01. This includes Sidak's amendment test for multiple comparisons.

**Number of embryos with WT or MUT phenotype**		**Replicate 1**		**Replicate 2**		**Replicate 3**		**Overall**		**Statistics**
		**WT**	**MUT**	**WT**	**MUT**	**WT**	**MUT**	**WT**	**MUT**	
**Trans** MO 4nM (n=153)	**KD**	2 (3%)	60 (97%)	14 (30%)	33 (70%)	4 (9%)	40 (91%)	20 (13%)	133 (87%)	
**Trans** MO 4nM + mRNA 25pg (n=207)	**RESCUE**	27 (40%)	41 (60%)	38 (61%)	24 (32%)	27 (35%)	50(65%)	92 (44%)	115 (56%)	**0,043 ***
**Splice** MO 4nM (n=147)	**KD**	13 (27%)	36 (73%)	2 (3%)	58 (97%)	2 (2%)	36 (0.95%)	17 (12%)	130 (88%)	
**Splice** MO 4nM + mRNA 25pg (n=157)	**RESCUE**	19 (42%)	26 (58%)	32 (53%)	28 (47%)	22 (42%)	30 (58%)	73 (46%)	84 (54%)	**0,0039 ****

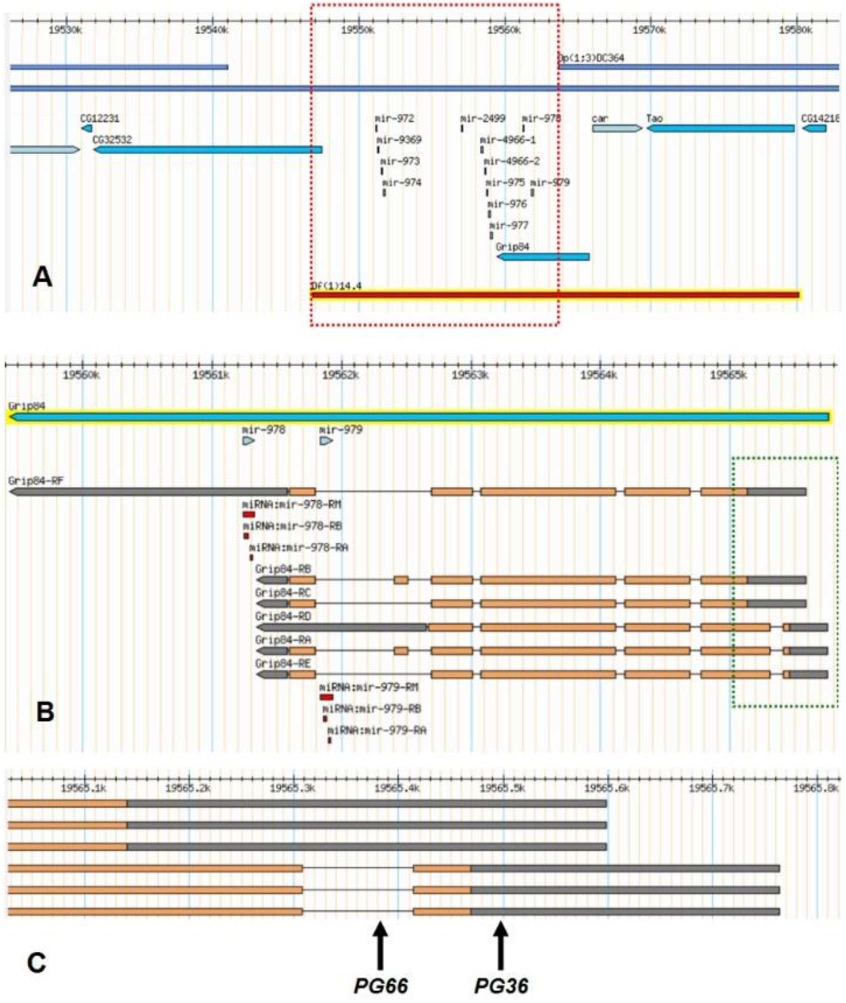

Supplementary Figure S1. Organisation of the *Grip84* gene in *Drosophila melanogaster*. (**A**) Genomic region surrounding the gene, showing a small deficiency, *Df(1)14.4* and left part of the duplication *Dp(1;3)DC364*. In the stock including both the deficiency and the duplication, the region in a red dashed rectangle is missing, therefore *Grip84* should be eliminated. Micro RNAs and *cg32532* are also eliminated. (**B**) structure of the gene, including alternatively used exons, the fragment in the dashed, green rectangle is magnified in (**C**) together with precise positions of two P elements.
